# A Hybrid Intelligent Framework to Combat Sophisticated Threats in Secure Industries

**DOI:** 10.3390/s22041582

**Published:** 2022-02-17

**Authors:** Danish Javeed, Tianhan Gao, Muhammad Taimoor Khan, Duaa Shoukat

**Affiliations:** 1 Software College, Northeastern University, Shenyang 110169, China; 2027016@stu.neu.edu.cn; 2 Riphah Institute of Science and Engineering, Islamabad 44000, Pakistan; taimourkhan86@gmail.com (M.T.K.); dua.shaukut@ptcl.net.pk (D.S.)

**Keywords:** Industrial Internet of Things (IIoT), software-defined networking (SDN), deep learning (DL), intrusion detection system (IDS)

## Abstract

With the new advancements in Internet of Things (IoT) and its applications in different sectors, such as the industrial sector, by connecting billions of devices and instruments, IoT has evolved as a new paradigm known as the Industrial Internet of Things (IIoT). Nonetheless, its benefits and applications have been approved in different areas, but there are possibilities for various cyberattacks because of its extensive connectivity and diverse nature. Such attacks result in financial loss and data breaches, which urge a consequential need to secure IIoT infrastructure. To combat the threats in the IIoT environment, we proposed a deep-learning SDN-enabled intelligent framework. A hybrid classifier is used for threat detection purposes, i.e., Cu-LSTMGRU + Cu-BLSTM. The proposed model achieved a better detection accuracy with low false-positive rate. We have conducted 10-fold cross-validation to show the unbiasdness of the results. The proposed scheme results are compared with Cu-DNNLSTM and Cu-DNNGRU classifiers, which were tested and trained on the same dataset. We have further compared the proposed model with other existing standard classifiers for a thorough performance evaluation. Results achieved by our proposed scheme are impressive with respect to speed efficiency, F1 score, accuracy, precision, and other evaluation metrics.

## 1. Introduction

The Industrial Internet of Things (IIoT) connects physical machines, sensors, and devices with the Internet. It then uses various software to perform deep analytics and transform vast amounts of data into powerful insights and intelligence [1]. This term highlights the IoT and its applications in sectors such as the Industrial sector, with strong attention on machine-to-machine (M2M) communication, machine learning (ML), and big data. Things covered in this domain include connecting wastewater systems, electric meters, flow gauges, manufacturing robots, other connected systems and industrial devices. With IIoT, enterprises and industries have better reliability and efficiency in their work [2,3]. The connecting working ability of multiple devices with the Internet allowes different threat actors to perform anomalous activities. There are a growing number of vulnerabilities and loopholes in the protocol used by IIoT architecture that threat actors can breach using sophisticated attack approaches [4,5]. An attacker’s motives behind the exploit are to gain valuable information, money theft, and to corrupt the resources [6]. By the end of 2030, cyberthreats could cost up to USD 90 trillion to the IIoT if no promising solution is presented until then [7,8]. With the rapid increase in connecting IoT devices, securing critical assets and infrastructure is becoming a serious concern for various businesses. With all this, IoT brings three challenges: the first one is the IoT’s heterogeneous network [9,10]. The second is its massively dispersed architecture, whereas the third is the protocols that IoT introduced for issues such as computation limitation and power in network sensors. In environments such as IIoT, the most common threat is Zero-day vulnerability leveraged by malware [11,12]. The attacker’s main objective is to infect the critical devices to obtain control and change their operations using various techniques such as Distributed Denial of Service (DDoS), Advanced Persistent Threats (APT), and Denial of Service (DoS) attacks. For example, in 2010, the Iranian Nuclear Program was attacked by Stuxnet Worm. After that, in 2013, Iranian hackers got into the ICS of New York’s Dam. In 2015, 230,000 customers in Ukraine suffered from a power outage due to black energy malware [13]. Hence, these occurrences proved that traditional cybersecurity procedures are no longer effective, including the authentication, security policies, firewall, and Intrusion Detection System (IDS). We propose an intelligent, SDN-enabled framework for timely and effective threat detection in IIoTs. The experimentation is conducted using the N-BaIoT dataset.

### Contribution

- We propose a novel SDN-enabled Intillegent framework for early and efficient threat detection in the IIoTs.
- Cu-LSTMGRU + Cu-BLSTM hybrid model is used for effective threat detection.
- We compare the performance of the proposed model with current benchmark algorithms, i.e., Cu-DNNLSTM and Cu-DNNGRU, trained and evaluated on the same dataset.
- For further performance evaluation, we compare the proposed model with existing literature.
- We have employed standard evaluation metrics for a thorough evaluation.
- Finally, 10-fold cross-validation is employed for verification purposes of our results.

The remaining paper is arranged as follows. In Section 2, we discuss the background and existing work. Section 3 is about the proposed methodology, dataset, and other details. Section 4 is dedicated to experimentation and evaluation criteria. Section 5 is about the experimental results, while the conclusion is discussed in Section 6.

## 2. Background and Existing Literature

SDN appears to be the most favorable networking model to be used in the coming years. The architecture of SDN comprises a data plane, control plane, and application plane with their APIs, i.e., northbound API and southbound API. The interface of northbound refers to the domain of protocol-based communication between the controller and applications or higher-layer control programs. Communication with the switch fabric, network virtualization protocols, and the integration of a distributed computing network are all functions of southbound APIs. According to SDNs architecture, we have a control plane isolated from the application and data plane. The control plane provides a review of the underlying basic network and is a centralized and intelligent unit. Apart from this, the control plane is a centralized decision-making and data-processing unit. Further, it has the potential to forward data to the whole network. However, the data plane represents the SDN agents and forwarding devices’ collection. The control plane is programmable, and it has the ability to enhance its functionality by implementing different modules as the entire framework depends on the control plane. Hence, SDN provides flexibility and innovation, and its detailed architecture is presented in [14,15]. All SDN controllers are capable of extending different modules. Because of this, the detection scheme proposed by the authors is implemented on the control plane. For different SDN controllers, the architecture and design for most of them are the same; however, they differ in functionalities. From controller to controller, the implementation language differs. For example, Java is the implementation language of floodlight, while Python is used for writing POX.

The deep-learning models have aided the area of computer science through their applications, which are used in almost every sector of business; from medical devices to autonomous vehicles. The models of DL use the architecture of neural networks, which is why these model are referred to as deep neural networks. These models use a large set of labeled data for training, that automatically extracts features from data without the requirement for manual feature extraction. Some other applications of DL are voice recognition, fraud detection, image classification, and threat detection, and it is also used for the detection of pedestrians which results in a decrease in accidents.

The contemporary scientific evolution has witnessed the manifested competencies of the Internet of Things (IoT) that encompass every facet of our lives. The conveniently acquirable nature of IoT makes it impressionable to a diverse domain of security threats that need to be addressed. Software-Defined Networks (SDN) are an imperative evolutionary technology that provide promising solutions toward the security and integrity of IoT. Several scientific contributions have been made to overcome the susceptible nature of IoT; however, SDN-based security solutions prove their effectiveness at pre-eminent ranking [16]. SDN also interacts with other relevant cutting-edge technologies to efficiently play the role under contention. The integration of SDN and blockchain is, presented which comprises all the crucial security concerns regarding IoT in a futuristic perspective. Preservation against Denial of Services (DoS) attacks, spoofing attacks, and routing attacks are the core aptitude of that amalgamation [17]. SDN-enabled security solutions are considered to be marvelous in terms of resource utilization. The constitutional scheduling mechanism of the SDN central controller always comes with remarkable management of network resources. Hence, SDN-enabled intrusion detection schemes inherit that feature and facilitate IoT in gratifying protection frameworks, disbursing the least possible resources [18]. Another security model needs to be mentioned here that is formulated to insulate sensitive IoT environments against a broader range of potential security threats. The proposed model consists of an SDN-enabled blockchain-inspired approach for large-scale receptive atmospheres. The performance of the concerned model is evaluated, where favorable results seem to make it an ideal choice for large-scale IoT networks [19]. SDN also shakes hands with convolutional neural networks (CNN) to equip a distinguished safeguard for IoT against the wide variety of legitimate concerns. The Distributed Denial of Services (DDoS)-based attacks tree is an alarming sign against the smooth flow of communication in an IoT-based automated environment. This phenomenon caught researchers’ attention, resulting in the designing of an SDN-enabled CNN-based security framework for resource-constrained IoT networks. The most significant feature of the proposed framework is efficient detection of security threats with less consumption of network resources [20].

In recent years, researchers have put their remarkable interest in deep learning and its applications in different research areas such as automotive designs, law, and the health sector. Moreover, lots of work exists in the area of NIDS in SDN [21]. A DL-based intrusion-detection framework was proposed by authors in [17], and employed RBM (Restricted Boltzmann Machine) in SDN. For experimental setup, this scheme used the KDD99 dataset and CMU dataset. For binary classification, this technique achieved 99.98% accuracy. Another scheme proposed in [18] utilized IDS based on GRU–RNN (Gated Recurrent Unit–Recurrent Neural Network) with CICIDS2017 and NSL-KDD datasets. The results showed an accuracy of 89% for different classifications. Although SDN architecture is flow based, the dataset NSL-KDD which was used is not flow-based. For attacks and threat detection in SDN networks, authors in [17] presented a DL (deep learning) system in which multilayer perception (MLP) is used. This scheme used the CTU-13 dataset, and performance results showed 98.7% detection accuracy. A connection-based technique is referred to as Credit-Based Threshold Random Walk (CB-TRW). Further, the authors implemented rate limiting in [19] with intrusion detection and prevention systems. For experimentation, network traffic was captured for five minutes. The results showed that false positive rate (FPR) is 0% with 97% CPU utilization for captured traffic of 10,000 packets at the rate of one second. In [20,21], the authors used the RNN, CNN, and LSTM for the network intrusion-detection framework. This framework used the ISCX2012 dataset. The model achieved an accuracy of 98%. The authors of [22,23] employed GRU-RNN for network intrusion detection systems (NIDS). The authors used the NSL-KDD dataset with six basic features. Results showed that the framework achieved an accuracy of 89%, which is not good for current evolving cyberattacks and threats. Authors in [24,25] proposed a method of anomaly detection entirely based on deep learning. This system used CNN, LSTM, and MLP. For experimentation, data were collected via T-Shark and Wireshark.

Authors in [26] proposed a DL method on a DNN for flow-based intrusion. This framework used Snort (network intrusion-detection system) with Barnyard and achieved 85% detection accuracy. Further, authors in [27,28] used a diverse variety of classifiers based on machine learning (ML) and a DL model. The authors used extreme learning machine (ELE), Ada-Boost, support vector machine (SVM), and decision tree. The authors proposed an intelligent intrusion-detection System (IDS) in SDN, using the dataset NSL-KDD, and acquired 80% detection accuracy. To address the issues of the Botnet detection mechanism, authors in [29,30] presented a scheme in SDN, which depends on multilayer perception (MLP). For experimentation, real data were used with an achieved accuracy of 98%. The authors in [31,32] presented an IDS using RNN and this IDS was trained by using the NSL-KDD dataset. The evaluation was performed on the network traffic. This model achieved 81.29% of accuracy for the classification of multiclass. Authors in [33] presented an SDN-based, intelligent scheme for intrusion detection in IoT. The authors used the CICIDS2017 dataset for training and experimentation using deep-learning classifiers and achieved a better detection accuracy. The literature review is summarized in Table 1.

## 3. Proposed Methodology

The purpose of this research is to propose an intelligent DL-driven scheme for threat detection in IIoT environments. This section is dedicated to the methodology of our work, i.e., hybrid threat-detection framework, preprocessing of dataset, proposed network model, and dataset description.

### 3.1. Proposed Network Model and Detection Scheme

**Figure 1:**
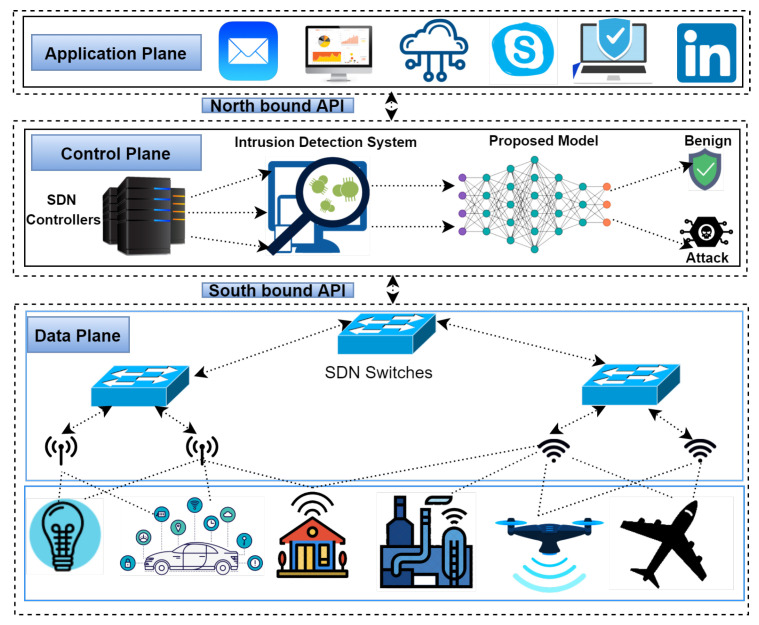
Network Model.

During the past several years, SDN has emerged as an integrated network design. The SDN’s application plane is designed to run a variety of applications in order to provide different services to endusers. The application mechanisms, on the other hand, are managed by the SDN’s control plane, which handles data transfers, routing decisions, and traffic monitoring. For simplification and flexibility purposes in the SDN design, the data plane and control plane are separated. In addition to this, the control plane came up with the network’s global view and central control functions, which simplified the assembling of network statistics. For the environment of IIoT, we proposed hybrid DL-driven, SDN-enabled architecture to detect threats and intrusion. Figure 1 depicts the proposed model (Cu-LSTMGRU + Cu-BLSTM) which is placed in the control plane of SDN. There are many reasons for establishing the proposed model in the control plane. First, it is completely programmable, and also it can extend the IIoT devices on the data plane. Second, open flow switches are used in SDN, which is the solution for heterogeneity among IIoT devices and SDN controllers. Furthermore, without any exhaustion, the control plane can manage the main devices of IIoT in its data plane. The data plane is in charge of forwarding actual IP packets and to transport data packets from the source to the destination. The SDN framework and IIoT incorporation propose a better way to deeply examine the network traffic to look for intrusions, unauthorized events, and attacks, with the advantage of being cost-effective and centralized.

**Figure 2:**
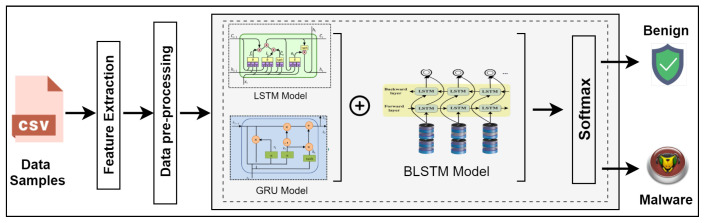
Detection Scheme.

Further, the authors propose a DL-driven hybrid model, i.e., Cu-LSTMGRU + Cu-BLSTM, for threat detection in IIoT. To detect various threats, a very powerful, versatile, and cost-effective scheme is developed that is visualized in Figure 2. This scheme comprises Cu-LSTMGRU and Cu-BLSTM models for sophisticated malware detection in the IIoT environment. The N-BaIoT dataset is tested and trained on the hybrid algorithms of deep learning with high detection rates and fewer false positives (FP). This scheme comprises multiple layers, i.e., Cu-LSTMGRU consists of 200 neurons and Cu-BLSTM has 100 neurons in one layer. We have used softmax in the output layer for the activation function and Relu function for other layers. For better results, the experimentation has been performed with 32 batch sizes until five epochs. We have used the Cuda-enabled version for experimentation purposes for faster multiplication of matrices.

Moreover, the proposed scheme uses the backend of Tensor flow and Keras framework for Python. By making use of the two classifiers, a comparison is made with the proposed scheme. The comparison classifiers are deep neural network–long short-term memory (DNN–LSTM) with one layer of DNN and LSTM comprising 200 and 100 neurons, respectively, and deep neural networks–gated recurrent unit (DNN–GRU), with one layer of DNN comprising 200 neurons and the GRU with 100 neurons as the other layer.

In addition to this, a comparison of our hybrid model is made with existing models, and the results are depicted in Table 6. By multiplication of matrixes, the whole performance of the system improves. In Table 2, an in-depth description of our DL classifiers is presented. However, the pseudocode of the proposed model is also provided as Algorithm 1.
**Algorithm 1** Hybrid cuLSTMGRU–cuBLSTM detection model1:**procedure**     **Input**: n th iiot features and malware labels:2:${X_{n}}^{iot},\;{Y_{n}}^{iot}$3:cuLSTMGRU layers = M; cuBLSTM layers = l; k-Folds = k; epochs= e;     **Output**: Get the Error E and predictions P.4:    Get the Error E and predictions P.5:    **for** ∀ k :=1 to 10 **do**6:        **for** (epochs :=1 to e **do**7:           **if** select.layer [M] = cuLSTMGRU **then**8:               Calculate update gate for timestamp t.9:               Calculate reset gate to determine how much of past information to forget.10:                Starting with the usage of reset gate, new memory content which will use reset gate to store information.11:                Calculating ht-Vector which holds information of the current position.12:           **else**13:               Generate a feature vector.14:           **end if**15:           **if** select.layer[l] = cuBLSTM **then**16:               Randomly generate the w and b of BLSTM17:               Compute the Hidden layers of BLSTM18:               Compute the output of Hybrid GRULSTM-BLSTM19:           **end if**20:        **end for**21:    **end for**22:**end procedure**

### 3.2. Dataset

For the evaluation of threat-detection scheme performance, the use of an appropriate dataset significantly matters. For threat detection in the IIoT environment, the literature review shows that different authors used different datasets, e.g., NSLKDD [43,44,45], KDD CUP99 [46,47], etc. Most of them do not have the IIoTs supportive feature. For IIoT devices, some attackers scan them and then take control of these devices. In addition to this, they also use DNS rebinding and malicious scripts for locating and attacking the IIoT devices. Hence, the dataset used for the proposed model is a publicly available dataset N-BaIoT [48]. This dataset constitutes the network flow and IIoT supportive features, and it comprises the most dangerous malwares, i.e., Bashlite and Mirai. It consists of eight attacks and up to 115 traffic features. The dataset instances distribution is presented in Table 3 below.

### 3.3. Preprocessing of DataSet

The proposed work performed the preprocessing of the dataset by the following steps. At the first step, we detected all the blank rows, rows with nan values, and then deleted all of them as they can impact the performance of the evaluation model and data quality. During the next step, using the label encoder, i.e., sklearn, we converted all non-numeric values into numeric values as mostly numeric data can be processed by DL algorithms. In addition, to diminish the chances of unexpected results, we executed one-hot encoding on the output label as model performance can also be reduced due to category ordering. Minmax Scaler is used for the purpose of data normalization, which enhances the model’s effectiveness.

## 4. Experimental Setup

For experimentation purposes, we used graphic processing unit (GPU) and Core i7-7700. Moreover, we used Keras to train the proposed module with a 3.8 version of Python. In Table 4, the software and hardware specifications are given.

### Evaluation Metrics

Using the standard evaluation metrics such as precision, recall, accuracy, and F1-score, we evaluated the performance of the proposed architecture. For certain parameters’ calculation, we have to calculate the false omission rate (FOR), true positive (TP), false positive (FP), true negative (TN), false negative (FN), and Matthew’s correlation coefficient (MCC).

(1) $$Accuracy=\frac{TP+TN}{TP+TN+FP+FN}$$

(2) $$Recall=\frac{TP}{TP+FN}$$

(3) $$Precision=\frac{TP}{TP+FP}$$

(4) $$F1-score=\frac{2*TP}{2*TP+FP+FN}$$

## 5. Result and Discussion

This section presents the complete results of the proposed hybrid model (Cu-LSTMGRU + Cu-BLSTM). For detailed performance evaluation, we compared this model with two other hybrid models, Cu-DNN–LSTM and Cu-DNN–GRU, along with existing techniques in the literature. The following standard metrics of evaluation evaluate the performance of the proposed model.

### 5.1. Roc Curve Analysis

**Figure 3:**
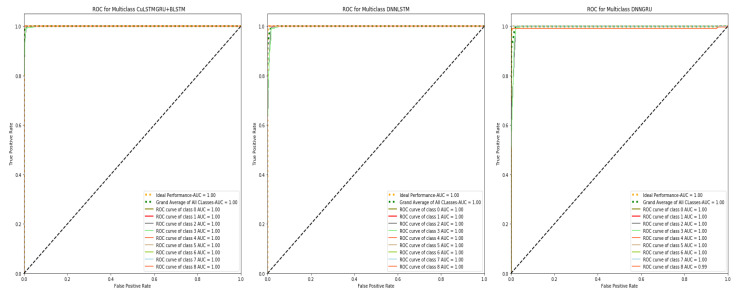
ROC curves of the models.

The Roc is a key parameter for checking the performance of any intrusion-detection system (IDS). True negative rates (TNR) and true positive rates (TPR) are correlated, and Roc plots the results. The Roc curve of our scheme is given below in Figure 3. This figure depicts the relationship between a true negative and a true positive.

### 5.2. Confusion Matrix Analysis

**Figure 4:**
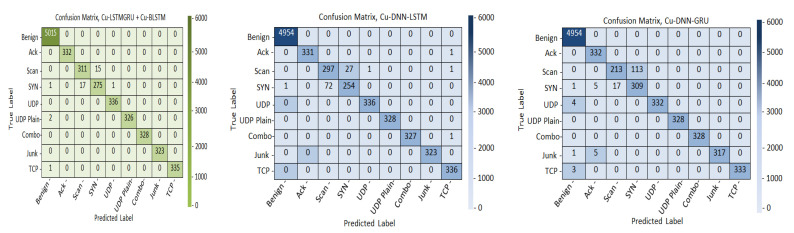
Confusion metrics of the models.

This evaluation matrix show the output of the classification model. As per the confusion matrix results, Cu-LSTMGRU + Cu-BLSTM recognizes the classes accurately. The confusion metrics of the three models are given in Figure 4. It depicts that the proposed model correctly identifies the classes and surpasses the other two models, (Cu-DNN–LSTM and Cu-DNN–GRU).

### 5.3. Cross-Validation

We used 10-fold cross-validation to prove the neutrality of our results. A detailed description of each fold is given in Table 5.

### 5.4. Accuracy, Recall, F1-Score, and Precision

**Figure 5:**
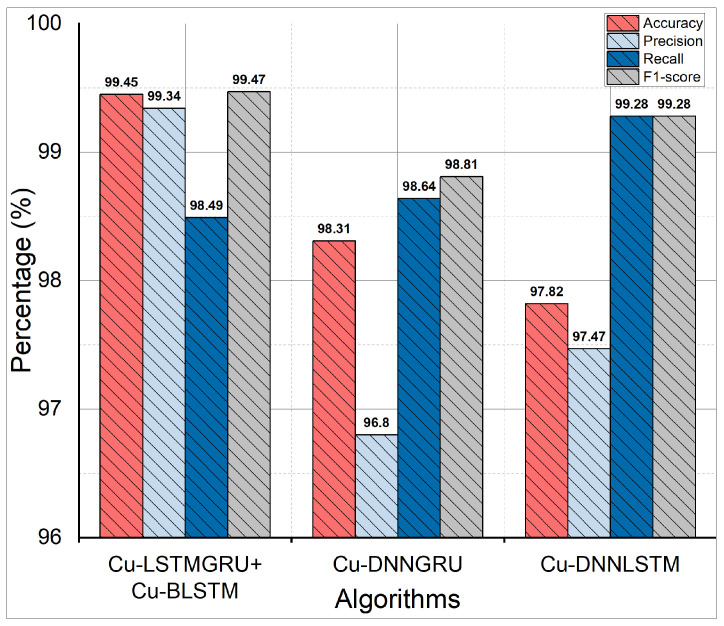
Accuracy, recall, F1-score, and precision.

The efficiency and performance of a classifier are demonstrated by accuracy. It shows how many samples are accurately identified by the proposed scheme. In Figure 5, we presented the accuracy performance of our proposed scheme (Cu-LSTMGRU + Cu-BLSTM). This hybrid model achieves 99.45% accuracy with 98.49% of recall. The records which are identified correctly indicate precision. The proposed model has a precision of 99.34% with a 99.47% F1 score. The 10-fold results are depicted in Table 5 for recall, precision, accuracy, and F1-score.

### 5.5. FPR, FOR, FNR, and FDR Analysis

**Figure 6:**
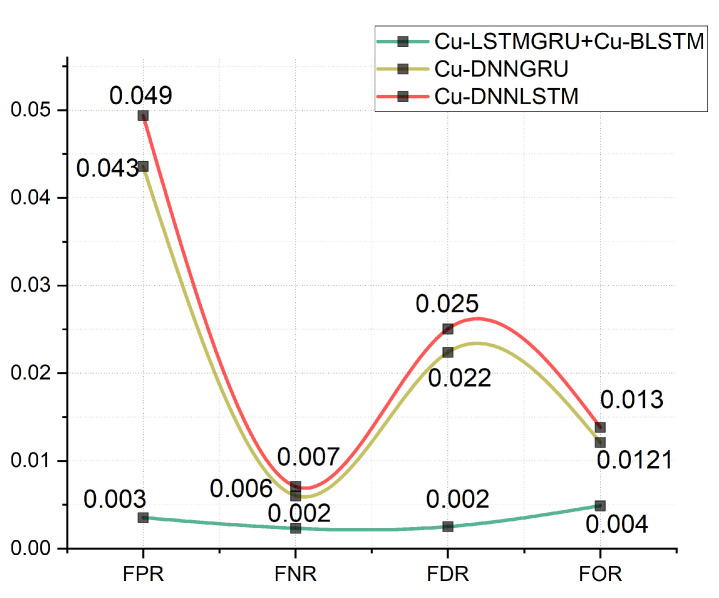
FPR, FNR, FDR and FOR Results.

To effectively evaluate our proposed scheme, we calculated the FOR, FPR, FDR, and FNR. The results are presented in Figure 6. We can see that our proposed model has FOR and FPR of 0.004% and 0.003%, respectively, while the FDR and FNR values are 0.002% and 0.0020%. Hence, our proposed model Cu-LSTM-GRU outperforms the other two models. In addition to this, DNN–GRU performs better than DNN–LSTM.

### 5.6. TPR, TNR, and MCC Analysis

**Figure 7:**
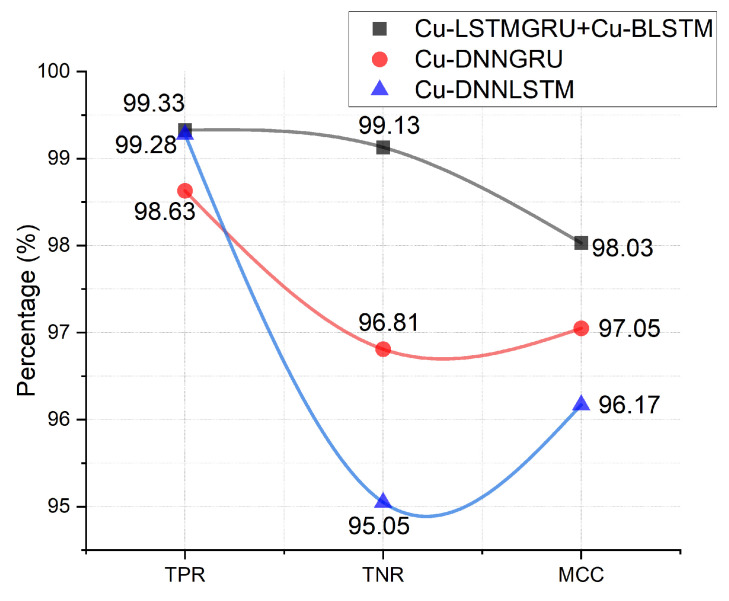
TPR, TNR, and MCC.

To evaluate further, we used a confusion matrix for in-depth analysis of the proposed model to obtain the TPR, TNR, and MCC analysis values. In Figure 7, TNR, TPR and MCC are shown with values of 99.33%, 99.13%, and 98.03%, respectively. By casting an analytical look at the Figure 7, it is concluded that Cu-LSTMGRU+Cu-BLSTM has better performance.

### 5.7. Speed Efficiency

**Figure 8:**
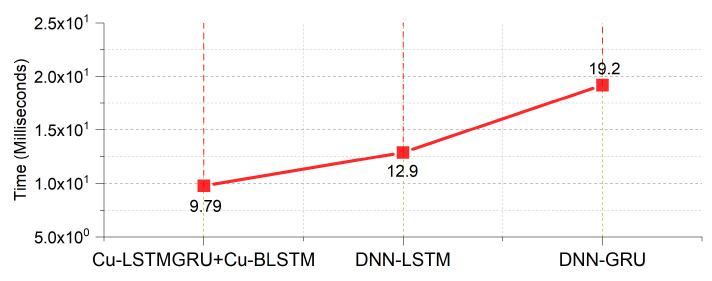
Speed efficiency of the models.

The time taken by the proposed model for testing is shown in Figure 8. Here, we are not considering the training phase as it was mostly performed offline. While illustrating the model’s performance and efficiency, testing is very important. The time consumed by our proposed hybrid model is 9.79 ms, which is computationally efficient. However, for the other two models, DNNLSTM is computationally better than DNNGRU, with a testing time of 12.9 ms.

### 5.8. Cu-LSTM-GRU–Cu-BLSTM Comparison with Existing Literature

To highlight the efficacy of the proposed scheme, we compared it with two existing hybrid DL models (Cu-DNN–LSTM and CU-DNN–GRU). For evaluation, we used the same metrics for both models and all of the three models were tested and trained on the same dataset N-BaIoT. The details of these models are given in Table 2.

Moreover, a comparison is also made with other benchmark algorithms. In Table 6, the proposed model’s comparison with the existing literature is given. It can be seen that Cu-LSTMGRU + Cu-BLSTM outperforms in terms of precision, F1-Score, accuracy, and speed efficiency. Furthermore, the testing time of the proposed model is 9.79 ms, which is significantly better than the existing benchmarks.

### 5.9. Limitations of the Proposed Model

The proposed hybrid model is a potential intrusion-detection system in an IIoT environment. Despite the considerable performance of our proposed method, there are some limitations that we will address in the future, i.e., the proposed model requires well-labeled data for training. On the other hand, these data are infrequent, and obtaining them necessitates a significant amount of effort. Further, the proposed intrusion-detection model outperformed the existing techniques; however, it will be more effective if it can detect insider attacks where intruders can harm the network without affecting the traffic flow between the sensor network and the internet.

## 6. Conclusions

There is a need for flexible and secure IIoT infrastructure. This can be achieved using Cuda-enabled deep-learning classifiers. Intrusion-detection systems based on DL can have the ability to detect any emerging cyberthreats. We proposed SDN-enabled, intelligent architecture to protect the IIoT environment from sophisticated threats. For successful threat detection, we have used a hybrid classifier (Cu-LSTMGRU + Cu-BLSTM). The proposed scheme is scalable, and also it has a low cost. Moreover, we compared the results with other hybrid algorithms, i.e., Cuda-DNNLSTM and Cuda-DNNGRU. Results showed that our proposed scheme outperforms the other two hybrid models and those existing in the literature. We have used standard evaluation metrics to evaluate the model, i.e., speed efficiency, F1 Score, accuracy, precision, recall, TPR, FPR, etc. The proposed scheme consumes a testing time of only 9.79 ms with 0.0035% FPR and 99.45% accuracy. Our model has better results as compared to the existing literature. In the future, the authors aim to use different hybrid classifiers along with blockchain and SDN for efficient threat detection, and will propose a scheme for isolating the compromised IIoT devices. Lastly, the authors endorse SDN-based intelligent frameworks for the security of IIoT environments.

## Figures and Tables

**Table 1:** Existing literature.

Ref	Year	Algorithm	Dataset	Achievements	Limitations
[7]	2019	SVM, RBM	CMU, KDD99	Proposed detection scheme for multiclass using SVM and RBM with an accuracy of 89%.	Dataset is not flow-based, old, and static.
[25]	2018	LSTM, CNN, RNN	ISCX2012	In the proposed scheme, feature filtration is performed with a verification accuracy of 98%.	Time overhead as the scheme is computationally complex.
[29]	2019	CNN, LSTM, MLP	Tools Tshark, Wireshark data	Used Fast Gradient Sign method (FGSM), JSMA, JSMA-RE to solve port scanning issue.	Computationally complex.
[32]	2018	DT, ELM, SVM, NN, Ada-Boost	NSL-KDD	For SDN proposed anomaly detection scheme with the detection accuracy of 80%.	Real-time environment performance of the classifier is not enough.
[34]	2019	MLP	CTU-13 ISOT	To detect botnet in SDN intrusion detection scheme is proposed based on MLP.	Experimentation is not performed on botnet infected terminals.
[35]	2019	MLP	Real time	Botnet detection scheme using MLP with a detection accuracy of 98%.	Evaluation is performed only on real-time traffic.
[33]	2019	RL, CB-TRW	Real traffic	In a software-defined network, DoS and port scan detection and prevention method is presented using RL and CB-TRW.	Only false-positive rate (FPR) and CPU consumption is used as a performance parameter.
[36]	2017	RNN	NSL-KDD	R2L and probe detection using RNN classifiers.	Comparison is made with machine-learning algorithm.
[37]	2021	DNNGRU-BLSTM	CICIDS2018	Obtained efficient detection rate by using a hybrid classifier of DL for multi-class attacks.	The proposed method cannot detect the DDoS attacks by reflecting all of the features of the blocks formed when the attack occurs.
[38]	2018	GRU-RNN	NSL-KDD	Using six network features, the proposed scheme GRU-RNN achieved 89% detection accuracy.	The dataset NSL-KDD is not flow-based.
[39]	2018	DNN	Barnyard	Proposed deep-learning and flow-based detection scheme with snort with a detection accuracy of 85%.	Computationally complex.
[40]	2012	Genetic Algorithm	KDD99	Obtained sufficient detection rate.	The dataset is not IoT-based and outdated, with high false-positive rates.
[41]	2018	RBM	KDD99	The authors achieved a precision rate of 94 %.	The dataset is not IoT-based and too old.
[42]	2018	CNN-RNN	CTU13-ISOT	The model can detect botnets at the packet level.	The detection accuracy is low, and time complexity is high.
[43]	2018	DM, SM	NSL-KDD	Achieved efficient output by developing shallow and deep models.	The dataset is not IoT based.
[44]	2015	SVM	NSL-KDD	Better detection accuracy.	Inherent limitations, the strong signal needed in data.
[45]	2018	LSTM-GRU	NSL-KDD	Achieved an accuracy of 87%.	The detection accuracy is too low.
[46]	2017	FLS-Based Approach	NGIDS-DS	Showed the rational attack activities and usual traffic changing aspects of real-world networks.	The complexity of the dataset is not explored properly.
[47]	2019	GRU-RNN	NSL-KDD, CICIDS17	Achieved 89% accuracy for multiclass using GRU-RNN classifier.	Diverse features are not used for enhancement of classifier.

**Table 2:** Hybrid algorithms description.

Algorithm	Layers	AF	Neurons	LF	Optimizer	Batch-Size	Epochs
	Cu-LSTMGRU (1)	Relu	(200)				
	Cu-BLSTM (1)	Relu	(100)				
Cu-LSTMGRU+Cu-BLSTM	Dropout	–	(0.3)	CC-E	Adamax	32	05
	Output Layer (1)	Softmax	07				
	Dense (3)	–	(200,100,50)	–			
	DNN Layer (1)	Relu	(200)				
	LSTM Layer (1)	Relu	(100)				
Cu-DNN–LSTM	Dropout	–	(0.3)	CC-E	Adamax	32	05
	Dense (3)	–	(200,100,50)	–			
	Output Layer (1)	Softmax	07				
	DNN Layer (1)	Relu	(200)				
	GRU Layer (1)	Relu	(100)				
Cu-DNN–GRU	Dropout	–	(0.3)	CC-E	Adamax	32	05
	Dense (3)	–	(200,100,50)	–			
	Output Layer (1)	Softmax	07				

**Table 3:** Dataset description.

Attack Category	Subcategory	Attack Instances
Benign	–	49,500
	Ack	3400
	Scan	3300
Mirai	SYN	3300
	UDP	3400
	UDP Plain	3300
	Combo	3300
Bashlite	Junk	3300
	TCP	3400
Total	–	76,200

**Table 4:** Experimental setup.

CPU	7700, i7, 7th Generation with 2.80 GHz processor
RAM	16 GB
GPU	Nvidia GeForce 1060 6 GB
Language	Python, version 3.8
Libraries	Keras, Numpy, Pandas, TensorFlow and Scikitlearn
OS	Windows 10, 64 bit

**Table 5:** 10-fold results of the hybrid models.

Parameter	Hybrid Models	1	2	3	4	5	6	7	8	9	10
Precision (%)	*Cu-LSTMGRU+Cu-BLSTM*	98.30	99.85	98.76	99.81	99.83	99.21	99.65	99.93	98.41	99.67
	Cu-DNN–LSTM	98.92	98.52	93.77	96.23	98.94	97.53	95.69	98.29	97.51	99.37
	Cu-DNN–GRU	97.76	96.50	95.30	96.50	96.50	97.40	96.90	96.90	97.15	97.10
Recall (%)	*Cu-LSTMGRU+Cu-BLSTM*	99.83	98.52	99.23	97.74	98.39	99.11	97.52	97.29	98.44	98.92
	Cu-DNN–LSTM	99.49	99.39	99.93	99.81	99.31	99.41	99.91	97.96	99.09	98.54
	Cu-DNN–GRU	99.37	98.50	98.50	99.30	99.30	99.37	98.30	98.21	98.21	97.37
Accuracy (%)	*Cu-LSTMGRU+Cu-BLSTM*	99.50	99.11	99.23	99.74	99.39	99.66	99.25	99.29	99.44	99.92
	Cu-DNN–LSTM	98.96	98.63	95.62	97.32	98.85	97.97	97.01	97.51	97.74	98.62
	Cu-DNN–GRU	99.18	97.73	95.64	98.36	98.81	99.23	98.94	98.31	98.85	98.10
F1-Score (%)	*Cu-LSTMGRU+Cu-BLSTM*	99.83	99.52	99.23	99.74	99.39	99.11	99.25	99.29	99.44	99.91
	Cu-DNN–LSTM	99.49	99.39	99.93	99.81	99.31	99.41	99.91	97.96	99.09	98.54
	Cu-DNN–GRU	99.37	97.80	97.50	97.70	99.20	99.15	99.40	99.40	99.10	99.50

**Table 6:** Comparison with existing benchmarks.

Ref	[47]	[49]	[50]	Proposed
Algorithm	GRU-RNN	Autoencoder(EDSA)	Multi-CNN	Cu-LSTMGRU +
				Cu-BLSTM
Dataset	CICIDS17	CICDDoS2019	NSL-KDD	N-BaIoT
Accuracy	89%	98%	86.95%	99.45%
10-fold	-	-	✓	✓
Multiclass	✓	✓	-	✓
GPU-Enabled	-	-	-	✓
F1-Score	99%	-	88.41%	99.47%
Recall	99%	-	87.25%	98.49%
Precision	99%	-	89.56%	99.34%
Testing time	-	-	-	9.79 ms

## Data Availability

Not applicable.

## Abbreviations

The abbreviations used in this paper are as follows

IoT	Internet of Things
IIoT	Industrial Internet of Things
MLP	Multilayer Perceptron
IDS	Intrusion-Detection System
LSTM	Long Short-Term Memory
ROC	Receiver Operating Characteristic
DDoS	Distributed Denial of Service
GRU	Gated Recurrent Unit
SDN	Software-Defined Networking
Cu	Cuda
DNN	Deep Neural Network
RF	Random Forest
API	Application Programming Interface
SVM	Support Vector Machine
CNN	Convolutional Neural Networks
RBM	Restricted Boltzmann Machine
FGSM	Fast Gradient Sign Method
ELE	Extreme Learning Machine
CB-TRW	Credit-Based Threshold Random Walk
GPU	Graphics Processing Unit
TP	True Positive
FP	False Positive
TN	True Negative
MCC	Matthew’s Correlation Coefficient
CPU	Central Processing Unit
TCP	Transmission Control Protocol
AF	Activation Function
OF	Open Flow
NIDS	Network Intrusion Detection System
APT	Advanced Persistent Threats
RELU	Rectified Linear Unit
RNN	Recurrent Neural Network

## References

[1] Tange K., De Donno M., Fafoutis X., Dragoni N. (2020). A Systematic Survey of Industrial Internet of Things Security: Requirements and Fog Computing Opportunities. IEEE Commun. Surv. Tutor..

[2] Al Shorman A., Faris H., Aljarah I. (2019). Unsupervised intelligent system based on one class support vector machine and Grey Wolf optimization for IoT botnet detection. J. Ambient. Intell. Humaniz. Comput..

[3] Mrabet H., Belguith S., Alhomoud A., Jemai A. (2020). A Survey of IoT Security Based on a Layered Architecture of Sensing and Data Analysis. Sensors.

[4] Haller S., Karnouskos S., Schroth C., Domingue J., Fensel D., Traverso P. (2009). The Internet of Things in an Enterprise Context. Proceedings of the Future Internet—FIS 2008.

[5] Bhunia S.S., Gurusamy M. Dynamic attack detection and mitigation in IoT using SDN. Proceedings of the 27th International Telecommunication Networks and Applications Conference (ITNAC).

[6] Ben-Asher N., Gonzalez C. (2015). Effects of cyber security knowledge on attack detection. Comput. Hum. Behav..

[7] Garg S., Kaur K., Kumar N., Rodrigues J.J.P.C. (2019). Hybrid Deep-Learning-Based Anomaly Detection Scheme for Suspicious Flow Detection in SDN: A Social Multimedia Perspective. IEEE Trans. Multimed..

[8] Xia W., Zhu W., Liao B., Chen M., Cai L., Huang L. (2018). Novel architecture for long short-term memory used in question classification. Neurocomputing.

[9] Tharwat A. (2020). Classification assessment methods. Appl. Comput. Inform..

[10] Koroniotis N., Moustafa N., Sitnikova E., Turnbull B. (2019). Towards the development of realistic botnet dataset in the Internet of Things for network forensic analytics: Bot-IoT dataset. Future Gener. Comput. Syst..

[11] Kim J., Kim J., Kim H., Shim M., Choi E. (2020). CNN-Based Network Intrusion Detection against Denial-of-Service Attacks. Electronics.

[12] Ghorbani A.A., Habibi Lashkari A., Sharafaldin I. Toward Generating a New Intrusion Detection Dataset and Intrusion Traffic Characterization. Proceedings of the 4th International Conference on Information Systems Security and Privacy 2018.

[13] Acar G., Huang D.Y., Li F., Narayanan A., Feamster N. Web-based Attacks to Discover and Control Local IoT Devices. Proceedings of the 2018 Workshop on IoT Security and Privacy, Budapest Hungary 2018.

[14] Al-Rubaye S., Kadhum E., Ni Q., Anpalagan A. (2019). Industrial Internet of Things Driven by SDN Platform for Smart Grid Resiliency. IEEE Internet Things J..

[15] Du M., Wang K. (2019). An SDN-enabled pseudo-honeypot strategy for distributed denial of service attacks in industrial Internet of Things. IEEE Trans. Ind. Inform..

[16] Alam I., Sharif K., Li F., Latif Z., Karim M.M., Biswas S., Nour B., Wang Y. (2020). A Survey of Network Virtualization Techniques for Internet of Things Using SDN and NFV. ACM Comput. Surv..

[17] Shukla N., Gandhi C., Choudhury T. (2021). Leveraging Blockchain and SDN for Efficient and Secure IoT Network. Blockchain Applications in IoT Ecosystem.

[18] Mazhar N., Salleh R., Zeeshan M., Hameed M.M., Khan N. R-IDPS: Real-time SDN based IDPS system for IoT security. Proceedings of the IEEE 18th International Conference on Smart Communities: Improving Quality of Life Using ICT, IoT and AI (HONET).

[19] Islam M.J., Rahman A., Kabir S., Karim M.R., Acharjee U.K., Nasir M.K., Band S.S., Sookhak M., Wu S. (2021). Blockchain-SDN based Energy-Aware and Distributed Secure Architecture for IoTs in Smart Cities. IEEE Internet Things J..

[20] de Assis M.V.O., Carvalho L.F., Rodrigues J.J.P.C., Lloret J., Proença M.L. (2020). Near real-time security system applied to SDN environments in IoT networks using convolutional neural network. Comput. Electr. Eng..

[21] Javeed D., Gao T., Khan M.T. (2021). SDN-Enabled Hybrid DL-Driven Framework for the Detection of Emerging Cyber Threats in IoT. Electronics.

[22] Wu K., Chen Z., Li W. (2018). A novel intrusion detection model for a massive network using convolutional neural networks. IEEE Access.

[23] Molina Zarca A., Garcia-Carrillo D., Bernal Bernabe J., Ortiz J., Marin-Perez R., Skarmeta A. (2019). Enabling virtual AAA management in SDN-based IoT networks. Sensors.

[24] Saharkhizan M., Azmoodeh A., Dehghantanha A., Choo K.K.R., Parizi R.M. (2020). An ensemble of deep recurrent neural networks for detecting iot cyber attacks using network traffic. IEEE Internet Things J..

[25] Li C., Wu Y., Yuan X., Sun Z., Wang W., Li X., Gong L. (2018). Detection and defense of DDoS attack–based on deep learning in OpenFlow-based SDN. Int. J. Commun. Syst..

[26] Vinayakumar R., Soman K.P., Poornachandran P. (2017). Evaluation of recurrent neural network and its variants for intrusion detection system (IDS). Int. J. Inf. Syst. Model. Des. (IJISMD).

[27] Schueller Q., Basu K., Younas M., Patel M., Ball F. A hierarchical intrusion detection system using support vector machine for SDN network in cloud data center. Proceedings of the 2018 28th International Telecommunication Networks and Applications Conference (ITNAC).

[28] Nguyen T.D., Marchal S., Miettinen M., Fereidooni H., Asokan N., Sadeghi A.R. DÏoT: A Federated Self-learning Anomaly Detection System for IoT. Proceedings of the IEEE 39th International Conference on Distributed Computing Systems (ICDCS).

[29] Huang C.H., Lee T.H., Chang L.H., Lin J.R., Horng G. (2019). Adversarial Attacks on SDN-Based Deep Learning IDS System.

[30] Torres P., Catania C., Garcia S., Garino C.G. An analysis of Recurrent Neural Networks for Botnet detection behavior. Proceedings of the IEEE Biennial Congress of Argentina (ARGENCON).

[31] Meng F., Fu Y., Lou F. A network threat analysis method combined with kernel PCA and LSTM-RNN. Proceedings of the 2018 Tenth International Conference on Advanced Computational Intelligence (ICACI).

[32] Latah M., Toker L. (2018). Towards an efficient anomaly-based intrusion detection for software-defined networks. IET Netw..

[33] Birkinshaw C., Rouka E., Vassilakis V.G. (2019). Implementing an intrusion detection and prevention system using software-defined networking: Defending against port-scanning and denial-of-service attacks. J. Netw. Comput. Appl..

[34] Maeda S., Kanai A., Tanimoto S., Hatashima T., Ohkubo K. A botnet detection method on SDN using deep learning. Proceedings of the 2019 IEEE International Conference on Consumer Electronics (ICCE).

[35] Letteri I., Del Rosso M., Caianiello P., Cassioli D. Performance of Botnet Detection by Neural Networks in Software-Defined Networks. Proceedings of the Second Italian Conference on Cyber Security.

[36] Yin C., Zhu Y., Fei J., He X. (2017). A Deep Learning Approach for Intrusion Detection Using Recurrent Neural Networks. IEEE Access.

[37] Javeed D., Gao T., Khan M.T., Ahmad I. (2021). A Hybrid Deep Learning-Driven SDN Enabled Mechanism for Secure Communication in Internet of Things (IoT). Sensors.

[38] Tang T.A., McLernon D., Mhamdi L., Zaidi S.A.R., Ghogho M. Deep Recurrent Neural Network for Intrusion Detection in SDN-based Networks. Proceedings of the 4th IEEE Conference on Network Softwarization and Workshops (NetSoft).

[39] Ujjan R.M.A., Pervez Z., Dahal K. Suspicious Traffic Detection in SDN with Collaborative Techniques of Snort and Deep Neural Networks. Proceedings of the IEEE 20th International Conference on High Performance Computing and Communications.

[40] Hoque M.S., Mukit M., Bikas M., Naser A. (2012). An implementation of intrusion detection system using genetic algorithm. arXiv.

[41] Dawoud A., Shahristani S., Raun C. (2018). Deep learning and software-defined networks: Towards secure IoT architecture. Internet Things.

[42] Pektaş A., Acarman T. (2018). Botnet detection based on network flow summary and deep learning. Int. J. Netw. Manag..

[43] Diro A.A., Chilamkurti N. (2018). Distributed attack detection scheme using deep learning approach for Internet of Things. Future Gener. Comput. Syst..

[44] Dhanabal L., Shantharajah S.P. (2015). A Study on NSL-KDD Dataset for Intrusion Detection System Based on Classification Algorithms. Int. J. Adv. Res. Comput. Commun. Eng..

[45] Dey S.K., Rahman M.M. FlowBased Anomaly Detection in Software DefinedNetworking: A Deep Learning ApproachWith Feature SelectionMethod. Proceedings of the 2018 4th International Conference on Electrical Engineering and Information Communication Technology (iCEEiCT).

[46] Haider W., Hu J., Slay J., Turnbull B.P., Xie Y. (2017). Generating realistic intrusion detection system dataset based on fuzzy qualitative modeling. J. Netw. Comput. Appl..

[47] Tang T.A., McLernon D., Mhamdi L., Zaidi S.A.R., Ghogho M. (2019). Intrusion Detection in SDN-Based Networks: Deep Recurrent Neural Network Approach.

[48] Abeshu A., Chilamkurti N. (2018). Deep Learning: The Frontier for Distributed Attack Detection in Fog-to-Things Computing. IEEE Commun. Mag..

[49] Sindian S., Samer S. (2020). An Enhanced Deep Autoencoder-based Approach for DDoS Attack Detection. Wseas Trans. Syst. Control.

[50] Li Y., Xu Y., Liu Z., Hou H., Zheng Y., Xin Y., Zhao Y., Cui L. (2020). Robust detection for network intrusion of industrial IoT based on multi-CNN fusion. Measurement.

